# “I Wasn’t Sure What It Meant to Be Honest”—Formative Research Towards a Physical Literacy Intervention for Preschoolers

**DOI:** 10.3390/children7070076

**Published:** 2020-07-13

**Authors:** Jonathan D. Foulkes, Lawrence Foweather, Stuart J. Fairclough, Zoe Knowles

**Affiliations:** 1 School of Sport & Exercise Sciences, Liverpool John Moores University, IM Marsh Campus, Barkhill Road, Aigburth, Liverpool L17 6BD, UK; 2 Physical Activity Exchange, Research Institute for Sport & Exercise Sciences, Liverpool John Moores University, 5 Primrose Hill, Liverpool L3 2EX, UK; L.Foweather@ljmu.ac.uk (L.F.); Z.R.Knowles@ljmu.ac.uk (Z.K.); 3 Movement Behaviours, Health, and Wellbeing Research Group, Department of Sport & Physical Activity, Edge Hill University, St Helens Road, Ormskirk, Lancashire L39 4QP, UK; Stuart.Fairclough@edgehill.ac.uk

**Keywords:** physical literacy, physical activity, preschool, children, early years, intervention, qualitative

## Abstract

Physical literacy (PL) as a concept is important in developing lifelong physical activity; however, there is little research exploring how PL can be developed during the preschool years. This two-phase qualitative study sought the insights of academics/expert practitioners and preschool staff towards PL in order to inform the design of future preschool PL interventions. Phase One comprised of nine semi-structured interviews with experts in the field of children’s physical activity and/or PL. Interview topics included perspectives on the concept of PL and recommendations for interventions targeted at improving preschool PL. Phase Two consisted of focus groups with practitioners from four local children’s centres. Focus groups explored perspectives on the feasibility and acceptability of proposed PL interventions. Interviews and focus groups were analysed by thematic analysis and means of representation, respectively. Findings revealed that whilst there was limited understanding about the concept of PL among preschool educators, knowledge of child development was evident and that all participants agreed that there was a need for further training for practitioners. Perceived barriers to promoting PL noted by practitioners included funding, policy, curricular priorities, parental opinions, and the preschool environment. It was recommended that interventions should be: (i) designed using a participatory approach including all key stakeholders, (ii) conducted over the long term, and (iii) incorporate opportunities for children to engage in free and outdoor play. Furthermore, any intervention should be flexible to allow for variation between children’s centres, aligned to current policy/children’s centre targets and provide training and resources in order to overcome perceived barriers.

## 1. Introduction

Physical literacy (PL) is defined by the International Physical Literacy Association (IPLA) as the “motivation, confidence, physical competence, knowledge, and understanding to value and take responsibility for engagement in physical activities for life” [1]. In recent years, PL has become increasingly relevant to physical education, physical activity (PA), and sports promotion internationally [2], and is widely understood to relate to an individual’s capacity for a physically active lifestyle [3]. PL has become a key focus of PA [2], with the concept of PL considered instrumental to the lifelong promotion of PA, learning and development, redefining how PA is understood, and stressing the importance of a holistic development of an individual’s physical potential [4]. However, given that PL is seen as an abstract construct, a lack of consensus is apparent regarding its conceptual underpinnings [3]. Recent research has identified the challenges in defining PL and its related constructs, highlighting misinterpretations and a distinct lack of consistency [5,6]. Currently there is little evidence to demonstrate the efficacy of PL interventions [2], or the best practice for supporting PL across the life-course. Without comparative data to provide evidence for best practice around the development of PL, current guidelines and policies can only offer vague guidance [7].

With evidence from the literature demonstrating that PA tracks over time during childhood [8,9,10], the preschool years are an ideal period to intervene and help support children’s PL towards lifelong engagement in PA. In England, an increasing number of children are attending preschools due to a government initiative offering 30 h per week of free early education [11]. These preschools are required to follow the Early Years Foundation Stage curriculum, which recently incorporated more holistic and PL aligned requirements for child development, including targets for not only physical development, but also personal, social and emotional development [12]. As such, and in accordance with the socio-ecological model of influences on behaviour [13,14,15,16], early years educational providers provide an opportune setting to support young children’s PL. In particular, preschool staff and teachers (i.e., educators/practitioners) are perhaps key social agents in supporting PL and shaping the PA behaviours of young children [17], while features of the physical environment within the preschool setting (e.g., outdoor space) may also influence PL and PA [18]. Understanding how preschool settings and staff can best support young children’s PL is important and such evidence can inform the development of appropriate PL programmes and interventions.

In order to support PL within preschool children it is important to conduct formative research to explore the thoughts, opinions, and practices of these key stakeholders, so as to inform the design and development of any possible PL intervention. This participatory community-based approach has previously been recommended as a method for increasing intervention effectiveness and involving key stakeholders in a sustainable way within preschool settings [19,20]. Due to the highly conceptual nature of PL the use of qualitative methods allows for greater depth and opportunity to elicit context in the data obtained. Studies in America [21] and Canada [22] used focus groups to elicit practitioners’ perceptions of children’s movement and learning and how PA could be improved during preschool hours, respectively. The studies reported that practitioners would benefit from staff training or workshops, additional equipment and resources, and increased funds for physical activity [22], and recommended that future interventions examine the impacts on children’s movement and learning of teachers moving with children during outdoor play and including more natural features in the design of outdoor play areas [21]. In a recent study, focus groups were used to explore parents’ and preschool practitioners perceptions of PA, and additionally, fundamental movement skill (FMS) competency, among English preschool children [17]. The study found that there was a need for more training for practitioners on how PA could be incorporated and delivered within the preschool setting, and it identified the home environment as needing to be supportive in promoting PA and FMS [17]. Whilst these studies examined practitioner views on PA and FMS, to date there has not been a study that has addressed PL alone and more specifically, how to inform training curricula and programme development. Furthermore, to the authors’ knowledge, no previous study has used a two-phase approach involving both experts and practitioners in order to develop a PL intervention for preschool children.

The aim of the present study is to explore the attitudes and perceptions towards PL among both experts from the field of child physical activity and health and preschool centre staff in order to help inform the development of future PL interventions for preschool children. This novel two-phase approach to informing intervention design will allow established academics from this field to put forth their ideas for best practice surrounding PL and intervention design, whilst also allowing preschool staff to offer their own opinions on PL, and subsequently their own thoughts and critique on the practicality and feasibility of recommendations put forth by the expert group during Phase One. 

## 2. Materials and Methods

### 2.1. Study Design

A two-phase approach was used for the study, with Phase One results used to inform Phase Two. Phase One consisted of semi-structured interviews with experts from within the field of children’s PL and/or physical activity and health. Whilst Phase Two consisted of focus groups with practitioners (early years educators) working in children’s centres (preschool settings). All interviews and focus groups were conducted by the lead author having received training from a co-author (ZK) with extensive experience of conducting qualitative research.

A phenomenological approach was used, in order to explore, describe, and analyse the meaning of individuals’ experiences [23]. For experts (Phase One), this included how prior experiences had shaped their views on PL and intervention design. For the practitioners (Phase Two), phenomenology allowed exploration of what it was like to “be them”, understanding their practice how they construct meaning and to understand this setting through which proposed interventions may be appropriate. Semi-structured telephone interviews (Phase One) and focus groups (Phase Two) were utilised within the study. The conversational nature of semi-structured interviews during Phase One allowed the interviewer to modify interviews as warranted by the responses or circumstances of the expert group interviewees, whilst making sure that relevant topics from the interview were covered [24]. Focus groups were used among practitioners in Phase Two in order to produce a dynamic, interactive discussion format [24], allowing the focus group facilitator to request clarification of participant responses and ask for additional information. Whilst this approach may have offered less confidentiality to participants than interviews, it was felt that participants would be more open to sharing their thoughts and opinions due to feeling supported through a sense of group membership [25].

### 2.2. Participants

Phase One participants were international experts (academics/experienced practitioners) within the respective fields of PL and/or physical activity and health, with expertise in early childhood. Purposeful sampling was triangulated between the authors in order to recruit individuals represented publicly online as working as senior academics or as practitioners/researchers. Academics were required to hold a senior position within a university (e.g., Senior Lecturer, Senior Researcher, or Head of Department), and to have published research in a relevant field. Practitioners/researchers were required to hold a position within a public or private organisation, at either local or national level, whose purpose was to increase children’s PL, physical activity, health, or sports participation. Twenty-one experts (twelve academics) were identified via online searches as meeting the inclusion criteria and were subsequently invited to take part in the study via publicly available email addresses. A participant information sheet, containing a consent form, was attached to the initial approach email, with participants requested to (digitally) complete and return to the lead researcher to confirm participation within a two-week timescale. There was no response from eight of the experts approached, with a further four agreeing to take part but unable to schedule a convenient time for interview. Therefore, nine “expert” participants took part in Phase One of the study (42.9% response rate; 6 female), made up of six academics (nationality: British, *n* = 2, Australian, *n* = 2, American, *n* = 2, Canadian, *n* = 1) and two practitioners (nationality: British, *n* = 2). 

Phase Two participants were educators/practitioners working within children’s centres (early education providers attached to primary schools) in Liverpool, Merseyside, UK. The centres approached had all previously taken part in previous research projects conducted by the authors. Seventeen children’s centre managers from across Liverpool were contacted via a publicly available email address, explaining the purpose of the study and requesting three to five members of staff responsible for teaching and learning activities with 3–5 year old children, to take part in a focus group. Initial emails included a participant information sheet providing details of the study and a gatekeeper consent form for the centre manager to complete and return (electronically). The participant information sheet noted that participating members of staff would receive a £20 shopping voucher for taking part in focus groups. Four centre managers agreed for their centre to take part in the study (24.0% response rate). Subsequently, centre managers received participant information sheets and consent forms via email, to be forwarded on to centre staff. In total 19 preschool staff (17 female) agreed to take part across four focus groups. 

Full ethical approval was obtained for this study from the University Research Ethics Committee at Liverpool John Moores University (reference number: 16/SPS/010). Full informed written consent was obtained from all participants prior to their participation in interviews/focus groups.

### 2.3. Data Collection and Analysis

#### 2.3.1. Phase One: Interviews

A semi-structured telephone interview was developed to explore experts’ opinions on the concept of PL and their perspectives on the design and development of a future intervention aimed at improving preschool children’s PL. Thematically, questions explored PL among preschool children, covering (in order): understanding of the term “physical literacy”; the physical environment and policy within children’s centres, training, intervention design, and barriers to PL. Questions were structured to flow naturally from one section to another, and to maintain the participant’s engagement in the interview process. Initial thematic questions such as, “What does it mean to you when experts/practitioners use the term “physical literacy?” were designed to put participants at ease before progressing on to more in-depth and challenging questions requiring them to draw on their own experiences and ideas for best/future practice. One week prior to interviews, participants were sent a confidential document with the study context and interview questions in order to help familiarise themselves with the questions and facilitate depth in participant responses to keep the interview efficient and reduce the burden on participants. Interviews were conducted between February and April 2016. A single trained interviewer (the first author) conducted interviews using Skype, with interviews lasting on average 45 min (range 00:32:39–01:13:14). Digital audio recordings were made of each interview (Call Recorder for Skype v1.2.69.1027, DVDVideosoft, Digital Wave Ltd, London, UK) and subsequently transcribed verbatim. Copies of interview transcripts were sent to participants for their approval prior to analysis being undertaken.

Phase One interview transcripts (221 pages, Ariel size 12, double spaced) were imported into NVivo v10 (QSR International, Burlington, MA, USA) for data handling and subjected to thematic analysis [26,27]. This process initially required the reading of individual transcript in order to assign broad thematic codes, several of which were pre-defined prior to interviews taking place, namely, defining PL, the preschool environment, programme design and implementation and training for practitioners. These broad codes were then subsequently organised into higher and lower order themes. Both inductive and deductive techniques were used to generate codes. Themes and associated codes were discussed with the research team using a reverse tracking process from codes to transcript. Verbatim quotations were taken directly from the interview transcripts in order to expand upon these themes within the summary table. Selected verbatim quotes were self-defining and self-delimiting and represented a single theme. For profile inclusion the threshold was set at a minimum of three participants in consensus of a particular theme, with themes/quotations of *n* = <3 deemed worthy of reporting presented within the subsequent results-based narrative or discussion. Phase One analysis outcomes were shown to an independent external researcher who had previous experience of qualitative research, bringing transparency, and data from interviews was reviewed by all three of the authors using a reverse tracking process from quotes within the summary table to the verbatim transcripts, allowing for alternative interpretations of the data [28]. 

#### 2.3.2. Phase Two: Focus Groups

A semi-structured focus group guide was developed to explore the perspectives of preschool staff on the feasibility and acceptability of future proposed PL interventions aimed at preschool children. The general dimensions of the focus group questions followed that of Phase One, with the same broad themes (understanding of the term “physical literacy”, the physical environment, training, intervention design and barriers to PL) covered in the same order to aid in flowing naturally from one section to another. The initial thematic question remained, and was only slightly re-worded to, “What did it mean to you when you heard the term physical literacy?” This question was designed to help stimulate discussion and interaction amongst the participants and help ease them into the format of the focus group before moving on to more in-depth questions where they would have to discuss their own working practices, details, and thoughts. Analysis of Phase One data highlighted prevalent ideas/thoughts on “best practice” for children’s centre staff that had been offered by the expert academics/practitioners. These ideas/thoughts were presented to children’s centre staff along with a verbatim quote for context, with children’s centre staff then asked for their opinions on these suggestions. For example, in regards to their centres current preschool environment, staff were asked if they felt PL could be improved by the provision of greater learning through play, mandatory outdoor play, mobile play equipment, and limited seating time. The basis for these categories was a consensus amongst experts/practitioners that these would bring about improvements in PL (see Table 1). Focus groups took place between June and July 2016, facilitated by a single trained researcher (lead author). Focus groups comprised on average five members of children’s centre staff (range 3–6 participants), with an average length of one hour and ten minutes (range 01:00:42–01:20:25), with all audio recorded using a digital Dictaphone and subsequently transcribed verbatim. Homogenous groups were used for the focus groups as it was important for participants to feel that they had similar views on the topics being discussed, making it easier to start a discussion [29] and having the advantage of participants being able to relate to each other’s comments on their shared experiences [30]. Additionally, homogenous groups were used as the aim of the focus groups was to look for a consensus in opinions among children’s centre staff. The participant information sheet advised participants that they would be allowed to bring in their own notes to the focus group and in doing so, for the researcher, this would help gain a greater depth in participant responses to questions. Participants were asked not to discuss the contents of these notes with other members of staff prior to the focus group taking place. One week prior to the focus group each centre received an email containing a two-page document, the first page containing a brief summary of the lead researcher’s previous research work and the second, a list of the questions that would be asked during the focus group. A flipchart was used during focus groups in order to help initiate discussion, with the facilitator writing down participant’s thoughts and ideas as they answered questions, and to aid participant recall and to clearly move discussion points forward from one section to another.

Representation of the Phase Two focus group data was completed via the use of verbatim quotes taken from the focus groups transcripts (202 pages, Ariel size 12, double spaced) to illuminate aspects of consensus making and emergent themes within the focus group discussions.

## 3. Results

Table 1 presents a summary of pre-defined higher order themes and representative quotes following coding, presented as percentage frequency for all Phase One expert participants. The most commonly coded higher order themes by frequency were Programme Design and Implementation (*n* = 209; 33.2%), Training for Practitioners (*n* = 166; 26.4%), The Preschool Environment (*n* = 155; 24.6%) and finally, Understanding Physical Literacy (*n* = 99; 15.7%). Table 2 presents verbatim quotes from practitioners that align with the higher order and emergent sub-themes from the expert group.

### 3.1. Understanding of Physical Literacy

#### 3.1.1. Expert Perspectives

Six participants were able to give, without hesitation, a clear and consistent definition of what they understood PL to be, namely, that PL consisted of a number of differing components and was not just limited to physical competency, aligning with the most recent definition put forward by the International Physical Literacy Association (IPLA) [1]. However, it was noted by a number of participants (*n* = 5) that there remains some confusion and misunderstanding as to the term “physical literacy”. The importance of PL was also raised (see Table 1), being described as an important foundation for lifelong physical activity (*n* = 4) and key to overall wellbeing (*n* = 3). Only two of the participants disagreed with the concept of PL and offered negative views, namely, that they were not “too convinced about the term [PL]” (Expert_A) and believing that the term itself “really is not new” (Expert_H). Two participants also commented on how they felt that a thorough understanding of the term was required at all levels in order for PL to be effectively implemented, as Expert_D stated: 

“I think a clarity between what physical literacy is in theory and practice, from root to branch, so from policy all the way down to on the ground practitioners, a knowledge and understanding of it [physical literacy].”

#### 3.1.2. Practitioner Perspectives

The initial responses from children’s centre staff during focus groups were that physical literacy was a term with which none of the participants were familiar. “Physical development, you hear constantly, but physical literacy, I’ve not really heard” (Participant1_CentreA). When participants explored the term further it was clear that whilst no clear definition of the term itself was offered, the literacy aspect of the definition led to a number of participants speculating that this was in some way the principal component of PL, through either the use of terminology or using PA to help improve children’s literacy, for example, “I initially took it to mean that doing physical activities actually enriched children’s language and understanding skills.” (Participant2_CentreA)

This self-referenced lack of knowledge surrounding the term “physical literacy” had led participants from each of the four centres to admit they had resorted to “Googling” the term “physical literacy” in order to gain a further understanding prior to the focus groups. 

At saturation point within the focus group, participants were then presented with the most recent IPLA definition of PL [1]. Children’s centre staff were all in agreement that this definition was positive and they supported the idea behind the concept and recognised that key aspects within it that would lead to a physically literate individual. However, whilst participants were broadly appreciative of the ideas put forward in the entirety of the IPLA definition of PL, they felt there were still issues with the translation of the definition itself to fellow staff and parents. The length of the definition was a concern for several participants, with it being described as “wordy” (Participant2_CentreA) and “long-winded” (Participant3_CentreD), with the recommendation that “it could be condensed” (Participant3_CentreA). Furthermore, focus group participants were unanimous in voicing their concerns that in its current form this definition was still difficult to understand, also expressing concern about using it to try and convey the concept of PL to parents noting, “because I don’t understand a lot of it [IPLA definition of PL] myself” (Participant4_CentreD). Two focus groups (Centres C and D) also reiterated that they would still struggle to get the concept of PL across to parents. One participant felt the only way of getting the message across would be if there was a “big push” on PL, similar to previous health-based initiatives, in order to attract people’s attention, such as “the Change for Life, it’s so simple...everybody can relate to that.” (Participant5_CentreB)

### 3.2. Policy and Environmental Strategies to Support Physical Literacy

#### 3.2.1. Expert Perspectives

Within the sub-theme of changes to policy (see Table 1), four lower order sub-themes relating to proposed changes in national policy were identified, with experts providing examples of changes they would like to see implemented in order to bring about improvements in PL. Namely, an increased importance of PL (*n* = 5), mandatory PL (*n* = 5), greater funding and research (*n* = 3), and mandatory physical activity (*n* = 3). Participants suggested a number of changes that could be made in order to help bring about a positive change in children’s PL, with four higher order themes identified as strategies to help increase PL (Table 1): learning through play (*n* = 5), limiting sitting time (*n* = 5), mandatory outdoor play (*n* = 4), and increased use of mobile play equipment (*n* = 3). 

Four lower order themes were further identified as barriers to supporting PL; a lack of understanding of the term “physical literacy” (*n* = 4), finance (*n* = 3), parents and family (*n* = 3) and physical space (*n* = 3). Two participants also highlighted preschool setting staff as a barrier and “the skill level of the staff and the lack of training that they’ve had” (Expert_F), with this lack of/access to training also noted by Expert_E, “They’ve [preschool staff] got a lot to teach, there’s lots of things on, and they don’t have the resources, they don’t have the money, they don’t have the access to professional development.” 

#### 3.2.2. Practitioner Perspectives

In terms of policies, two of the focus groups raised the issue that there would need to be a specific local or national government target set for PL before it could become a priority within centres, and more importantly in turn allow funding for PL. The issue of finances was raised by three of the focus groups, “Funding is so tight, we’ve [children’s centre] got to justify everything that we do.” (Participant1_CentreA).

Academic/practitioner suggestions for preschool polices and environmental strategies to help bring about improvements in PL (i.e., greater learning through play, mandatory outdoor play, an increase in mobile play equipment, and limited sitting time) were put in turn to the four practitioner focus groups. Increased learning through play was met with positive responses from all of the focus groups when asked if they felt this was appropriate, “absolutely” (Participant2_CentreC), “definitely” (Participant3_CentreC), with one participant adding “that’s their [children’s] way of learning anyway, especially in the early years” (Participant4_CentreC). Two of the focus groups discussed how they were already aware of the importance of this concept. For example, “Most of us are nursery nurse trained, so we’ve understood play for many years. Even our outside play outing is usually, large [gross] motor skills is always planned” (Participant3_CentreB). While a participant from a separate focus group noted the importance of setting staff understanding the importance of play as a learning opportunity for children (see Table 2). Three of the focus groups also raised the issue that it is important to get the concept of play as a learning opportunity for children across to parents. For example,

“See, I think we’ve got it [learning through play], because we’ve had it drummed into us…but I think it’s passing it on to the parents now when they go outside to play, they just stand and watch them, instead of getting involved. So it’s about teaching them now.” (Participant1_CentreD).

The idea of mandatory outdoor play for children was also met with a positive response from all focus groups, with staff saying that they would “agree” (Participant3_CentreC) with this idea, believing that “you should [have mandatory outdoor play]. I really think you should” (Participant1_CentreD), and that one participant also felt that outdoor play, “Can impact on behaviour as well. Some children need to be outdoors, and you can just see their behaviour improve” (Participant6_CentreB). However, one participant was quick to note the value of mandatory outdoor play would also have to be demonstrated to parents (see Table 2).

Whilst all of the focus groups supported the idea of mandatory outdoor play, participants were also able to highlight the difficulties in trying to implement this idea, with the issue of physical space with older nurseries for example, not having access to “continuous outdoor play” (Participant_CentreA). Further, all four focus groups were unanimous in saying that they felt there would be resistance from parents in this regard. For example, “They [parents] don’t take to it [bad weather] very well at all” (Participant3_CentreA). When asked to describe if they felt their current preschool physical environment was helpful in supporting children’s PL, only staff from Centre C expressed that they were happy with the current environment, elaborating, “I mean, we have got access to outdoor space. I mean, I’m personally happy with the environment. I think we’ve got everything we need, because the hall is so large, and because of the age of the children.” (Participant3_CentreC).

Conversely, the remaining focus groups felt that their current preschool environment was not helpful in improving children’s physical activity. A lack of both indoor and outdoor space was mentioned as being prohibitive to enhancing children’s PL in three of the centres (see Table 2). All four focus groups reported how they used external facilities such as church halls (Centre B) or a local park (Participant4_CentreC) for what they considered to be physical activity primarily due to the lack of space in their centre. Whilst participants from Centre B had access to nearby school’s facilities, these were not always available to them, due to being “hired out” to other organisations for activities such as “summer camp or summer school”, meaning the facilities were available during “term time only.” Despite the use of external facilities being an aid to centres, during two of the focus groups, staff were keen to note that the use of external facilities added a financial burden to the centre and were unsure whether this would be able to continue. For example, “and we have to pay for that [large external hall]. That comes out of our budget. So if any of our budgets get slashed again, then maybe that will have to go.” (Participant1_CentreD).

An increase in mobile play equipment was also seen as a positive by the four focus groups, with a number of focus groups giving examples of how much children enjoy using mobile equipment and the increased levels of activity they see with this type of equipment. For example, “When they [the children] can move things around and make stuff out of it, the concentration and the confidence is there, isn’t it, and they’ll stay for longer, and they’ll work co-operatively as well.” (Participant1_Centre A).

However, participants from three centres discussed how they also struggled with a lack of storage space for equipment. Centre B had to get rid of a large portion of their mobile play equipment due to no longer having the storage space for it and Centre D reporting they had the budget to buy additional equipment but nowhere to store it. 

When the issue of fixed equipment arose in follow up questioning, as opposed to mobile equipment, participants from one focus group talked about how they disliked fixed equipment “because I feel as though that [fixed equipment] doesn’t, children’s imagination, they can’t, sort of it stifles their imagination a little bit” (Participant3_Centre D). Another potential issue regarding fixed equipment discussed was that it may not be suitable for all age ranges. However, one of the focus groups were still particularly keen on fixed equipment and looking to invest in more (Centre C). 

The final suggestion for improving PL taken from the academic/practitioner group was the concept of limiting sitting time for children (see Table 1). The response from all of the focus groups was positive, with participants from one focus group stating, “Yes, definitely. I think some children need that [limited seating time] in classes” (Participant2_CentreB), and that children “need like little breaks, don’t they? Like little movement breaks” (Participant6_Centre B). Whilst one participant went so far as to say that limiting sitting time “should be mandatory” (Participant4_CentreD). One advantage of limiting sitting time that was raised by staff from three focus groups was that limiting seating time or movement breaks could help “get concentration to come back, and concentrate better” (Participant1_CentreB). As with other suggestions, there were participants who raised some concerns about how limited sitting time could be implemented, namely around the difficulties staff could face and how it might affect children differently, “Some children will sit and learn better sitting down in a place, but some children just won’t.” (Participant2_CentreA).

### 3.3. Programme Design & Implementation

#### 3.3.1. Expert Perspectives

Participants perspectives on PL programme design are presented in Table 1, with four sub-themes: collaboration (*n* = 6), buy in from staff (*n* = 4), activities and experiences (*n* = 4), and primary goals (*n* = 9) identified as key by interviewees. Additional sub-themes included the need for the inclusion of motor skills (*n* = 4), the adherence/engagement of centre staff (*n* = 3), physical activity (*n* = 3), and a range of environments (*n* = 3). Additionally, two participants felt it would be important to set goals for staff as well as for the overall programme, namely so that preschool staff could “improve their own confidence and competence in being able to implement structured activity” (Expert_F), and “Could feel confident and competent to deliver this successfully for the rest of their careers, and…continue to evolve their understanding [of physical literacy].” (Expert_E). 

Table 1 presents the programme components that experts would want to see within a PL intervention, with two higher order themes: intervention delivery and intervention duration and dosage. For intervention delivery, the majority of participants believed that preschool staff should be responsible for delivering an intervention (*n* = 5), with a degree of flexibility in the programme for it to work effectively (*n* = 3), and with unanimous agreement that any proposed intervention should not target children by sex (*n* = 9). The idea of an assessment tool being embedded into the programme was also suggested (*n* = 2) in order to assess children across time points, “It’s about having evaluation points all the way through” (Expert_G). Furthermore, the suggestion that an intervention would have to be “fun” was also put forward, “It’s got to be exciting, engaging, the children have got to be enthused, and it’s got to be fun, and that’s got to be for the children and the practitioner when they’re delivering it [proposed intervention].” (Expert_C).

The majority of participants felt that an intervention would have to be embedded into the current children’s centre curriculum in order to be successful (*n* = 6), with a number offering their views on minimum duration to provide a positive effect (*n* = 6), ranging from four-six weeks and up to a year. However, two participants advised that the longer an intervention ran for then there is the possibility that its effects may be diminished, for example, “We saw that [reduced effect of intervention over time], and this is with researchers, not classroom teachers or other trained professionals, but we saw that the more intervention time, the dose, that the intervention gets a little bit weaker.” (Expert_I).

#### 3.3.2. Practitioner Perspectives

Initially each focus groups was asked who they felt was best placed to design a PL intervention for young children. The consensus across the focus groups was that rather than being developed by an academic(s) the programme design should be a collaborative effort (see Table 2). Participants from two of the focus groups felt that parents “should be involved as well” (Participant1_CentreD) in the programme design for it to be successful, with one participant stating, “I think you’ve got to include parents, because…you really want it to start from there” (Participant3_CentreB). One area of consensus among all of the focus groups was that educating/engaging parents should be a primary goal of any intervention, for example, “Making them [parents] educated, making parents the educators. They’re ultimately responsible for their children’s physical development” (Participant2_CentreD).

Focus groups were then asked what they felt should be the goal(s) of an intervention aimed at improving the PL of children within their centre. Responses varied across the four groups, with suggestions ranging from wanting a programme to “help the children to be more school-ready” (Participant4_CentreB), to wanting to see a programme “make a difference to childhood obesity, or obesity in general, the family obesity” (Participant6_CentreB), whilst another focus group felt that they would be looking to try and encompass all aspects of PL, as well as place an importance on emotional wellbeing, “I think you’ve got to be in a good place to be able to think about eating healthy and doing exercise, so it’s about, for me, because I’m coming from that part, it’s about having good emotional wellbeing, feeling good, and then you’re able to do actually anything.” (Participant2_CentreC).

Focus groups were then asked to describe what activities or experiences they would like to see included in an intervention. Whilst one participant simply asked for “access to equipment and ideas of what to do with them” (Participant3_CentreA), three of the focus groups touched on the idea of wanting to have children do activities that were outdoors, or offered the opportunity to move in a different environment through access to swimming pools (Participant2_CentreB) or the park, “like a programme that you do in the park.” (Participant4_CentreD).

Similarly, one focus group talked about being able to provide more activities in the children’s centre through external practitioners, i.e., dancing (Centre B), which was something they had done previously. Participants in one of the focus groups suggested that they would like to see a broader range of sessions that could be applied to a range of children, stating, “It [session] needs to be specific, but groups for different ages as well” (Participant2_CentreD). The consensus among three of the four focus groups was that an intervention would be delivered most effectively by setting staff, seeing this as a “constant” with parents liking “to know who they’re coming in to” and that children like to “see that familiar face all the time” (Participant2_CentreD). Furthermore, one focus group dismissed the idea of using external practitioners due to cost (Centre D), whilst participants from another focus group (Centre B) felt that intervention delivery should be the responsibility of an external practitioner, as staff were already undertaking a number of roles, “We’ve just got so many hats on at the moment” and that an external practitioner would be able to provide “real energy and commitment” for any intervention being delivered. (Participant1_CentreB).

Although the consensus was that staff should deliver an intervention, the preschool staff were clear to point out that the appropriate training and additional support would still be required (further detailed below). When asked how long they felt an intervention would have to run for before a positive difference in PL could be observed, all of the focus groups felt that a long-term approach would be required. 

### 3.4. Training for Practitioners

#### 3.4.1. Expert Perspectives

Suggested training for preschool staff is presented in Table 1, with four sub-themes identified; child development and motor skill competency (*n* = 6), educational background (with a lower order sub-theme of lack of knowledge (*n* = 4)), understanding of physical activity (*n* = 3), and understanding of PL (*n* = 3). Further sub-themes relating to training for practitioners related to staff engagement (*n* = 6), resources (*n* = 3), online resources (*n* = 3), first-hand experiences (*n* = 3), and professional development (*n* = 3) available to preschool staff. All nine participants offered opinions on how they felt training should be delivered to staff, with further suggestions including time (*n* = 2), as training needs to be offered/carried out when it is convenient for preschools. For example, “It [training] really needs to fit at the right time, rather than it’s prescribed and it’s rolled out, and some people’ll take it, and some people don’t.” (Expert_G).

As well as positive feedback (*n* = 1) in order to help upskill and enable staff “to share good practice” (Expert_C) and goal setting (*n* = 1) so setting staff can set, “Much more ambitious and much more creative goals around physical activity for the kids.” (Expert_A). 

#### 3.4.2. Practitioner Perspectives

Focus groups discussed what training would be required for preschool setting staff in order for them to effectively improve children’s PL, starting with what skills or knowledge they felt were needed by staff. A range of responses were forthcoming from participants, with no definitive consensus. One participant discussed the importance of high-quality training and practical ideas for staff prior to delivering a session(s), noting that despite training “You think, ‘What am I supposed to be doing? I’ve got an hour here with ten two-year-olds. I need some ideas’ (Participant2_CentreD). Another focus group looked at training for staff who may not understand the concept of PL and therefore training “would encompass that” and “tell them why they need” (Participant3_CentreC).

Additionally, these same focus group participants believed that a broader training programme including basic physical activity and exercises would be beneficial for all and that “Just the basics of why it’s [physical activity] important” (Participant3_Centre C). One participant also described how they felt that for any training or knowledge to be effective then there had to be the support for it from centre managers who “need to be understanding the importance of it [training], so that it can cascade down to the staff members.” (Participant3_Centre D).

Questions then explored the specific details of how training for centre staff could be implemented. For some this encompassed a “variety of training approaches” (Participant3_CentreB) and there was no consensus amongst the group as to whether on site or off site would be best. One focus group noted that on-site training would be better for them, “If you did go out, would it be like one of us at a time, because you can’t let everyone go, because the centre’s got to still run” (Participant1_CentreD). Conversely, a differing focus group (Centre A) gave several reasons as to why they felt off-site training would suit them better, noting the benefits of a “mix of people coming…from all different centres, we’ve all got different spaces, and you get to share ideas that way as well.” (Participant2_CentreA).

With regard to onsite training, whilst a participant from one focus group felt releasing staff would not be an issue (Participant3_CentreC), other focus groups felt that this may be an issue, for example, “It kind of needs to come back with a package, because whoever goes on the training is going to have to then, if you like, share it with the others” (Participant1_CentreA). Participants mentioned difficulties in allowing staff to attend off site training sessions, one possible solution when asked was a train-the-trainer approach whereby a single member or small number of staff from a centre could attend a training session and would be subsequently responsible for training up other members of staff in their centre. Two of the focus groups also discussed how this was an approach to staff training that they had used previously or were currently using. Limitations to the train-the-trainer approach, namely possible time constraints were an issue for centre staff, “having that opportunity to come back and find the time to train up all the other staff, isn’t it, and having resources and materials?” (Participant1_CentreA), and that of only having one member of staff trained because “what if that member of staff leaves? You’re left then with nobody to train the trainers.” (Participant4_Centre D).

In respect of training content, two of the focus groups participants believed that there should be a practical element to training, for example, “It should be interactive, and you should be actually doing some of that physical activity” (Participant2_Centre A). In addition, three of the focus groups felt that training should incorporate some kind of follow up for staff and not simply be a one off. “It’s kind of having support in place that it can be implemented, but not in a way forced, but because people want it to happen, because they know it’s for the benefit.” (Participant3_CentreC).

Again, following on from Phase One of the study, whereby academics/practitioners had been asked the same questions relating to the knowledge/skills that preschool centre staff would require in order to improve PL (see Table 1), three suggestions were put to focus group participants, taken from the consensus opinions of the academic/practitioners. Focus group participants offered their views and opinions on the three areas of knowledge academics/practitioners had felt were most important, namely, child development and motor competence, an understanding of PL, and understanding the importance of PA. There was a positive consensus from all four focus groups to the recommendation that staff should have an understanding of child development. Some participants felt that staff within their centre already had this knowledge and understanding, with one participant was keen to point out that with the mandatory Early Years Foundation Stage (EYFS) Guidelines [31] that centres were now more aware of child development due to these mandatory guidelines, “but I think with EYFS [Early Years Foundation Stage], which obviously the nurseries are using anyway, if the child is delayed, that would be getting picked up, and obviously children have a two-year-old check as well, which is where most of our delays are picked up on, but it’s usually speech and language delay.” (Participant2_CentreB).

In response to the Phase One outcome that an understanding of PL would be required by centre staff (see Table 1), the four focus groups agreed with this and were open and accepting to the idea. These same issues were cited previously by the focus group, namely that “the term [physical literacy] is confusing” (Participant1_CentreA). The final academic/practitioner suggestion that staff should have an understanding of the importance of physical activity (see Table 1) was again met with a positive response by the focus groups. Additionally, one of the focus groups (Centre A) felt that there should be some form of follow up for staff, allowing them to monitor their own progression and receive further advice and support and for “someone coordinating it [the intervention], that keeps you in touch with the people you did your training with, doesn’t it, and keeps you in touch with whether you’re doing it right.” (Participant2_CentreA).

## 4. Discussion

The aim of this study was to explore the attitudes and perceptions towards PL from experts from within the field of child physical activity and health and preschool centre staff in order to inform the development of future PL interventions aimed at preschool children. Utilising a novel two-phase approach allowed the thoughts and opinions of experts within Phase One to inform the questions put forth to preschool centre staff in Phase Two, affording preschool centre staff the opportunity to provide a critique of the recommendations put forth by the experts within Phase One. The discussion will be broken down into sections addressing the key themes identified by both Phase One and Phase Two participants following the analysis of the semi-structured one-to-one interviews and focus groups, respectively.

### 4.1. Understanding Physical Literacy

The academic/practitioner expert group in Phase One and the children’s centre staff in Phase Two differed in their respective understanding of the term “physical literacy”. Among the academic practitioners there was a consensus of what it meant to be physical literate, in line with the definition put forward by the IPLA [1]. A recent review of definitions, foundations and associations of PL [5], reported 70% of the included studies referring to PL adopted this “Whiteheadian” definitional perspective. However, the term was unknown to all of the children’s centre staff that participated in the focus groups, who found the IPLA definition to be confusing and felt it was difficult for them to understand. The majority of experts noted how they felt there was still a great deal of confusion and that the term was “commonly misunderstood” (Expert_E). In order for the term PL to translate and be applicable to staff “in real world settings”, children’s centre staff suggested that a shorter definition to that of a memorable phrase could help to present the message more clearly. An example of this simplified approach to defining PL can be seen in the work undertaken by Sports Wales [32]. Sport Wales have produced their own simplified definition of PL as part of a large-scale initiative looking to promote PL. The Sport Wales definition is presented as a formula wherein “Physical skills + Confidence + Motivation + Lots of Opportunities = Physical Literacy.” [32], providing a number of resources incorporating this definition for families and professionals working with children from 0–16 years of age. In Canada, the Sport for Life Society have endorsed the IPLA definition of PL [1], but provide additional simplified descriptions of the three main components of PL, namely, motivation and competence, physical competence, and knowledge and understanding [33]. Similarly, Sport Australia offer a simplified definition, describing PL as being “about building the skills, knowledge, and behaviours that give us the confidence and motivation to lead active lives” and highlighting the physical, psychological, social, and cognitive skills domains within their PL framework [34]. Resources such as those produced by Sport Wales would seem to be helpful in raising awareness of the concept of PL to children’s centres, with the wider Physical Literacy Programme for Schools and Physical Literacy Framework being successful in raising awareness of holistic and authentic pedagogies and supporting whole school agendas relating to PL [35]. However, to date this approach has not been implemented across the rest of the United Kingdom. All four focus groups were aware of, and in agreement with, the requirements for PL within the current early years curriculum guidelines [31]. One possible solution for beginning to increase awareness of PL among children’s centre staff could be to provide PL resources such as those provided by Sport Wales alongside the national early years curriculum guidelines, such as the EYFS in the UK [31], making them easily available and translatable for children’s centre staff and parents/carers nationwide.

A further inductive theme amongst focus groups was the importance of practitioners being able to effectively convey the concept of PL to parents/carers as well as staff. Conversely, during Phase One discussions with academics/practitioners there was no mention of parental understanding of the term PL. Whitehead [4] states that during the early years parents or principle carers are the most significant individuals in the development of PL. The finding that parents/carers are important agents in supporting PL is consistent with previous studies reporting that positive parental behaviours can increase children’s FMS competency [36] and PA levels [37] and have been identified as being key to engaging children in PA and development of FMS competency [17]. The family/home environment is an important context where parents/carers influence their children’s PA behaviours via direct (e.g., provision of equipment, outdoor access, and independent mobility) and indirect (modelling behaviour and positive encouragement) actions [36,38]. This further highlights the need for “buy in” of the concept of PL, identified by experts during Phase One (see Table 1), to be extended to parents/family as well as children’s centre staff. As such, the findings of the present study would seem to indicate that it would be of value to researchers to consult with parents/carers on the design of future research projects/interventions aiming to develop children’s PL, in line with previous research that has recommended greater parental involvement is required in order to increase levels of both PA and FMS competency [17].

### 4.2. Barriers to Physical Literacy

The indoor and/or outdoor space available within children’s centres was identified as a barrier to PA (and in turn supporting PL) among both experts/practitioners and children’s centre staff and, accordingly, it was stated that a “one-size-fits-all” approach to intervention delivery would not work. Instead, an intervention may have to offer strategies that children’s centre staff can use to adapt to their current setting, making use of the facilities they have available to them. A practitioner noted that they “could do a lot in a confined space” (Expert_F) and had seen pre-schools and education settings where such practice had occurred. This belief that setting-appropriate planning and preparation could compensate for a lack of facilities was also put forward by participants in Tsangaridou [39] study among early childhood teachers. Likewise, it has been noted that a physically literate individual would have the ability to be physically active in a range of environments [40], further suggesting that the physical environment is a barrier that could be overcome through maximising a child’s opportunities to be active within a range of activities/experiences. Therefore, providing staff with activities/experiences within an intervention that could be implemented indoors or outdoors and adaptable to fit a variety of spaces may help to provide a solution to physical space being a barrier to improved PL.

Funding was also discussed as a barrier to PL. Whilst expert discussion of finance focused on funding for ideas such as the promotion of PL, children’s centre staff were more concerned with finances not being available to support new schemes or courses, with children’s centres nationwide having been affected by a reduction in funding within the UK [41]. Without a designated government target for PL, or a policy supporting its promotion, it would be difficult to prioritise PL within their centres or gain necessary funding. Whilst the present EYFS guidelines make reference to physical development [12], with stated targets/goals, PL is not explicitly mentioned. One solution to this problem may be to implement a PL intervention that would aid centres in meeting mandatory guidelines. The EYFS guidelines [31] include physical development and personal, social, and emotional development as focal areas for development during the early years, falling in line with the concepts outlined in the IPLA definition of PL [1]. Furthermore, whilst current UK PA guidelines [42] are not a target for preschools, supporting children’s PL during preschool would better prepare children to be more physically active when they reach compulsory school age. With the UK national PA guidelines [42] reviewed and revised in line with the latest scientific evidence [43], it is possible that future guidelines may provide specific PA targets for preschool children, possibly in line with the UK Governments most recent strategy to tackle childhood obesity, which includes the provision of a minimum of 30 min of PA per day while at school [44]. Alternatively, future preschool guidelines may begin to focus more on the differing activities throughout the day, e.g., physical activity, sedentary behaviour, and sleep, as opposed to one single component. This would be in line with Canada’s 24 h Movement Guidelines which integrate physical activity, sedentary behaviour, and sleep for children aged 0–4 yrs across the whole day [45], which has also been incorporated within the Australian 24-Hour Movement Guidelines for the Early Years (birth to 5 years) [46].

### 4.3. Strategies to Support Physical Literacy

Four suggestions for how to help support PL among children were compiled from the ideas put forward by academics/practitioners (i.e., use of mobile play equipment, learning through play, mandatory outdoor play, and limited sitting time). These suggestions were met with positive responses from the four practitioner focus groups, who agreed that these were strategies they would support and felt could contribute to increasing PL. However, whilst focus groups participants were supportive, they raised a number of potential issues regarding these suggestions that would need to be considered in the design of an intervention hoping to incorporate them successfully. Additionally, it is worth noting that all of the recommendations put forward by academics/practitioners were directly related to increasing PA, with little considerations for the other elements considered key to PL; motivation, confidence, physical competence, and knowledge and understanding. As such, this would indicate that further research is required in order to understand how best these other elements of PL could be promoted among preschool children and the role of preschool staff in helping to develop these competencies.

Whilst all focus groups were in agreement with the academic/practitioners that mandatory outdoor play would be complimentary to improving PL, the issue of physical space was again discussed in this context. Furthermore, as with the issue of defining PL, parents/carers were again seen as a potential barrier in regards to that of implementing mandatory outdoor play, with all four focus groups discussing how they had faced objections from parents/carers when wanting to take children outside in wet conditions. This is in line with previous research reporting restrictive behaviour from parents/carers resulted in reduced child PA [47,48]. With evidence showing that outdoor play is positively associated with PA and that children are more likely to move more outside compared to when they are inside [49,50], mandatory outdoor play could provide the opportunity for children to increase their PA levels and in turn enhance their PL. This could be especially important for children from areas of high deprivation, who are more likely to be exposed to neighbourhood and home environments that are limiting to PA due to increased neighbourhood safety concerns [51,52]. With evidence showing that the provision of school physical education can result in increased engagement in and sustainability of PA [53], it would appear that increasing the amount of time preschool children get to spend in outdoor play would be beneficial.

Focus group participants felt the suggestions of increased learning through play and limited sitting time could be effectively implemented within a preschool setting. Whilst a traditional didactic, academic, and content-based approaches to preschool education may come at the expense of a more child-centred, play-oriented, and constructivist approach to learning [54], changes to the preschool curriculum could provide children with further opportunities to progress on their PL journey. Although changes to the curriculum would require time to adapt via appropriate lesson plans or provide alternate teaching/learning tasks that could incorporate these approaches, there would be no additional financial costs for children’s centres. This approach has previously been trialed in Wales, with the implementation of a holistic play-based learning continuum for children 3–7 years old with specific subjects replaced by areas of learning, resulting in children who were independent, motivated, and active learners making good progress in the development of PL [55]. Increased learning through active play may also offer further opportunities for children to be active during the day, reducing the amount of time children spend in sedentary time and aiding in improving PA and FMS competency [56].

### 4.4. Training for Children’s Centre Staff

Whilst the majority of academic/practitioners believed that children’s centre staff require knowledge of child development and motor skills in order to help improve PL, many focus group participants felt that staff within their centres already had adequate knowledge and understanding. If the majority of children’s centre staff already have a basic understanding of child development then it may be that training for staff included as part of a PL intervention could focus more closely on other aspects, namely, physical competence, motivation and confidence, and knowledge and understanding [1]. With evidence that children’s motivation towards physical education and sport decreases with age [57], this indicates that children’s centre staff may need to ensure that children remain motivated towards PA during this young age. One way of helping to achieve increased motivation among children could be to provide lessons or activities that are task-oriented [58] or could draw upon the TARGET framework (Task, Authority, Recognition, Grouping, Evaluating and Time) put forward by Ames [59], in order to create a mastery-oriented climate for children, or by engaging in child initiated play, as detailed in the EYFS [31]. An intervention could again assist with this through the provision of resources for children’s centre staff including suggestions for guided learning plans and task-oriented activities.

Similarly, staff engagement and confidence was discussed as being an important factor within training delivery by the majority of the academics/practitioners. Evidence shows that in early childhood education there is a need to provide teachers with professional development opportunities [60,61], with professional development for preschool staff having a positive effect on curriculum and instruction [60,61,62,63]. To facilitate engagement from staff, training could seek to incorporate experiential learning in order for children’s centre staff to gain practical experience. Experiential learning approaches are unique in that they allow trainees an immediate opportunity to practice newly introduced or developed skills as well as providing them with immediate feedback about their performance [64]. In the physical education literature there are a number of examples of training that have utilised experiential learning as well as interactive sessions, on-site coaching and group reflection in staff training [65,66,67]. These approaches may appeal to children’s centre staff to address barriers identified in relation to staff training, namely difficulties in allowing staff to attend off site training sessions, providing the requested “variety of training approaches” (Participant3_CentreB) and delivering a sustainable train-the-train model of staff training.

### 4.5. Programme Design

Participants agreed that the design of a PL intervention should be undertaken as a collaborative process and that any intervention should be delivered by children’s centre staff. Participatory research is the co-construction of research between researchers and the population affected by the issue(s) being researched and/or the decision makers who apply research findings [68,69]. By incorporating the views and opinions of children’s centre staff during the design phase either through utilising findings of formative studies such as the present study, or through direct consultation with children’s centre staff, would allow elements of flexibility to be incorporated into the intervention, allowing an intervention to work on a “teacher’s terms” (Expert_D). Of note, is previous research that has used this design approach in order to develop successful interventions aimed at increasing preschool PA [19,20]. Including parents/carers in the intervention design may provide an opportunity to educate them on the concept of PL, an issue already previously raised by focus group participants given the role parents/carers have in shaping their child’s PA behaviours [70,71] and highlighted by Roscoe, James and Duncan [17], although their study was looking at PA levels and FMS competency as opposed to PL.

With regards to the duration and dosage of a proposed intervention, a number of academic/practitioners (*n* = 6) suggested minimum durations ranging from to six weeks to a year. Likewise, it was felt that an intervention should be embedded into the current curriculum of a children’s centre and become “part of the regular programme” (Expert_B). This long-term approach was also favoured by focus group participants, with suggestions that once an intervention was put in place “that (it) would just continue maybe” (Participant4_CentreC) or that it should just be “ongoing” (Participant1_CentreD). No previous studies have examined PL interventions in preschool children; however, looking at the elements of PL, the literature reports a number of different findings in relation to such intervention durations. In Gordon, et al. [72] meta-analysis of the effectiveness of PA interventions among pre-schoolers it was reported that interventions less than four weeks in duration had the largest effect on moderate-to-vigorous PA. However, the authors noted that the shorter duration resulting in the most effect may have been as a consequence of the intervention type, i.e., environmental changes, and not as a result of the duration of the intervention. Likewise, Morgan, et al. [73] systematic review and meta-analysis of FMS interventions in youth found that interventions ranged in duration from four weeks up to three years, with considerable variation in design as well as duration. These findings would seem to suggest that a longitudinal preschool intervention should be designed either through an initial programme of several weeks/months that could include elements that could gradually be embedded into a children’s’ centres curriculum, or designed to fit in with the current curriculum from the outset. A formative cooperative partnership on intervention design and implementation between researchers and children’s centre staff and other relevant stakeholders, e.g., parents, could help to address this and identify the best approach, ensuring the longevity of the programme and its continued development within a centre. Whilst an additional focus on evaluation led by researchers and incorporating the thoughts and opinions of centre staff/parents could be used to examine the long-term effectiveness and feasibility of the intervention.

### 4.6. Methodological Considerations

The strength of the present study is that it has actively sought to gain the views and opinions of both experts from within the field of children’s physical activity and health and children’s centre staff via a two-phase approach, allowing areas of dissonance and resonance between these two groups to be identified. Furthermore, the use of thematic analysis allowed for the portrayal of the consistent themes in the academic/practitioner group, avoiding minority views expressed to be overstated. With regards to limitations, a relatively low number of children’s centres agreed to take part in focus groups for Phase Two of the study (24% response rate). As such, whilst the views expressed in these focus groups do not represent the opinions of children’s centre staff across Liverpool or the UK, it has allowed for recommendations to be made for future preschool interventions within this area and beyond. Likewise, in Phase One there was a greater number of academics (*n* = 7) than practitioners (*n* = 2) who were recruited as experts; as such, the views of expert practitioners within this field may have been underrepresented. Additionally, it is possible that the participants from Phase One may have chosen to participate in the study due to their interest within this research area and thus may have introduced an element of bias into the findings. Unfortunately, no objective data was gathered in relation to the space available to each of the children’s centre. With the issue of physical space a constant issue this data could have added to the study as it would have been possible to examine whether focus groups who stated that space was an issue in their centre actually had more or less physical space in comparison to other centres. Additionally, with the importance of the role of parents/carers in the design/implementation of any PL intervention stressed by both experts and practitioners, it may be seen as a weakness of the study that parents/carers were not participants in some form.

## 5. Conclusions

Following the Phase One interviews and Phase Two focus groups a series of recommendations are presented to inform the design of a future intervention(s) aimed at improving PL among children:

1. The initial goal of a PL intervention should be to educate children’s centre staff about the concept of PL through onsite training prior to the start of the intervention, ensuring staff fully understand the concept, particularly elements relating to motivation, confidence, knowledge, and understanding. In turn, children’s centre staff will be able to cascade this concept to children’s centre staff and parents/carers through a train-the-trainer model and engagement in experiential learning.
2. An intervention should be designed in collaboration between researchers, children’s centre staff, and other relevant stakeholders who have the skills/knowledge to aid in the effective design and delivery of the programme.
3. There should be flexibility in the intervention design to allow for variation between settings, e.g., the physical space available, storage, or differing targets/priorities between centres.
4. Physical resources should be made available for the duration of the intervention and beyond for children’s centre staff, providing them with reference materials and ideas for activities that they can implement within their centre, e.g., session plans and activity cards.
5. The intervention should be aligned to current policy/centre targets in order to ensure that it will be feasible for centres to allocate time and resources, i.e., funding for the delivery of the intervention and aid in its continuation and sustainability over the long-term.

## Figures and Tables

**Table 1:** Summary of higher order themes and emergent sub-themes (minimum consensus of ≥3 participants from Phase One interviews with experts).

Higher Order Theme	Frequency (% Total)	Sub-Theme	Example Quote
Understanding of physical literacy	15.7	Lack of consensus/awareness	“My concern, is the message isn’t clear… what PL is, is not clear at all to schools that we work with and to pre-school settings.” (Expert_C)
		Importance of physical literacy for children	“I think of physical literacy as the foundation, or the core, or the building blocks to lifelong physical activity.” (Expert_B)
		Physical Competency	“Most of them [children] are below where they should be in terms of developmental milestones.” (Expert_C)
		Assessment of physical literacy	“I spent a couple of years trying to get people [researchers] to talk about measurement and how we would measure it [physical literacy], it was one of the most challenging things in my life.” (Expert_B)
Policy and environmental strategies to support physical literacy	24.6	Barriers to implementation	“Fragmentation, that it’s really difficult, everybody’s [children’s centres] kind of doing their own thing, and wanting to do their own thing, and it’s quite difficult to try to roll out something [intervention/programme].” (Expert_A)
		Barriers within preschool environment	“I think there’s quite a lot of differences [between centres], depending on the physical facilities where the nurseries are based, but also depending on what policies and what the sort of the thinking and the culture, I suppose, that’s cultured by the nursery teachers and the leads.” (Expert_A)
		Changes in national policy	“Ensure that it’s mandated through policy and curriculum documents that children need to be exposed to educative movement context and learning experience to develop their physical literacy.” (Expert_E)
		Changes to preschool environment	“Playground opportunities, high quality playground programmes or outdoor spaces for individuals would be great, in terms of environmental changes.” (Expert_I)
		Greater child focused learning through play	“I would get them [setting staff] to really understand and be confident in that active play is not frivolous, it is learning.” (Expert_B)
		Limited seating time for children	“The small things teachers can do, taking ten minutes out of your day… do some type of movement and physical activity break, or taking ten minutes after lunch, and ten minutes when they wake up from a nap. That’s thirty minutes right there [of physical activity].” (Expert_I)
		Mandatory outdoor play	“I think most daycares or pre-schools take their kids outside at some point, but I would highly suggest they do it more, and longer… but just go outside for an extra thirty minutes, an extra hour, every day.” (Expert_B)
		Mobile play equipment	“Kids are more active when there’s mobile play equipment for them… if there’s a tricycle, they’re more likely to use the trike, or balls or hoops or whatever the equipment is, if it can move, they’re likely to move it, or move with it.” (Expert_H)
Programme design and implementation	33.2	Collaboration between experts and practitioners	“I’d be sceptical of an academic designing an intervention that didn’t have the real-world experience, but still I’d be sceptical of an intervention designed purely for the real world that didn’t have the evidence base, so it has to be a combination.” (Expert_E)
		Buy in from centre staff	“It’s about getting buy-in…that’s the biggest issue at the moment. I’m looking at trying to elevate the value of physical literacy at the moment, so that people within school actually start to gain buy-in.” (Expert_D)
		Primary goal(s) for intervention	“Actual engagement in activity, both within the care setting, but also possibly at home.” (Expert_B)
		Intervention delivery	“I guess you can imagine a world in which we can just hire a bunch of specialists to go out and do this, but I just think the resources to do that are enormous, and I won’t speak for anywhere other than the US, but I just think that that’s not likely to be a successful approach.” (Expert_H) “Oh, it definitely has to be the settings staff [delivering an intervention], yes. It has to be the preschool educators.” (Expert_F)
		Duration and dosage of intervention	“I think for something to become embedded in a service, you probably need to have a longer period of time…. I think between twelve and eighteen months at least.” (Expert_F)
		Activities and experiences	“A graded variety of tasks that have different levels of challenge are really important, because kids, particularly young kids, will lose motivation and become easily and quickly frustrated if it’s too hard or they can’t do it.” (Expert_E)
		Targeting boys and girls separately	“No, I would support keeping them [both genders] together, or structuring the activity and the environment so that it isn’t perceived as a competitive activity, and that they can learn together and learn from each other.” (Expert_A)
		Outside of the preschool	“We can’t underestimate the impact that the family have on the children within the preschool setting. So we need to address that as well as preschool setting.” (Expert_C)
		Unlimited budget	“I would put an expert in every pre-school. I would put a full-time expert in every pre-school in the country, whose primary job was to get these kids active, playing games, moving proficiently.” (Expert_B)
Training for practitioners	26.4	Early years teacher training	“There’s a massive lack of impetus with physical literacy on initial teacher training and on childcare course…. I think people are coming away from their training as postgrads and achieving their certificates of childcare without a firm grasp of what physical activity and physical literacy really means.” (Expert_C)
		Understanding of physical literacy	“In terms of the knowledge and understanding that they [preschool staff] need, I think they need a basic understanding of what the concept [physical literacy] is initially.” (Expert_D)
		Understanding of physical activity	“The need to understand the importance of PL and of physical activity for our nation as a whole, and how it impacts on children’s lives.” (Expert_C)
		Child development and motor skill competence	“They [children’s centre staff] definitely need to have a basic component or basic understanding of child development, and I guess, motor skill competency.” (Expert_I)
		Expertise of external trainer	“I would have to say I would favour someone with a physical education background, or a kinesiology background for that matter, at the very least, but again, that also depends on the person.” (Expert_B)
		Training delivery	“I think that when you’re working with practitioners, they need to see the importance of what you’re [external practitioner] trying to do.” (Expert_F)

**Table 2:** Verbatim quotes from Phase Two focus group participants corresponding with higher order and sub-themes identified within Phase One interviews.

Higher Order Theme	Sub-Theme	Example Quote
Understanding of physical literacy	Lack of consensus/awareness	“It’s not something I’ve come across. Physical development, you hear constantly, but physical literacy, I’ve not really heard.” (Particpant1_CentreA)
	Importance of physical literacy for children	“You need all of those things [outlined in definition of physical literacy]. You need to be motivated, don’t you, and be confident, and actually competent as well [to be physically active].” (Particpant2_CentreA)
	Physical competency	“There is a delay in gross motor skills in our children in this area.” (Partcipant1_CentreB)
	Assessment of physical literacy	“We don’t, do we?” (Particpant1_CentreA) “I think we’re not great at measuring things, and we need to get better at that, and that’s something we’re working on at the moment.” (Participant2_CentreB)
Preschool environment and environmental changes	Barriers to implementation	“[A lack of] accessibility, and space, and resources, and money.” (Participant1_CentreD)
	Barriers within preschool environment	“We’ve only got a very small outdoor space in the children’s centre. It’s like a postage stamp. So there’s not much you can do.” (Participant2_CentreD)
	Changes in national policy	“Because with the pressures of the National Curriculum, and the testing children, this has put pressures on schoolteachers to reduce the number of hours that children are spending in physical activity, and I don’t necessarily agree with it, but I think that the more physical activity children have, then the better able they are to be able to do the academic side of their schooling.” (Participant1_CentreC)
	Changes to preschool environment	“We’d like a big outside area, and we’d like a big room. A hall would be nice.” (Participant2_CentreD) “[We’d like] a nice big outdoor space.” (Particpant1_CentreB)
	Greater child focused learning through play	“That’s their [children’s] way of learning anyway, especially in the early years.” (Partcipant4_CentreC)
	Limited seating time for children	“That should be mandatory.” (Participant3_Centre D) “I’d say no more than fifteen [minutes of sitting]. After twenty minutes, you can see the agitation generally, yes.” (Participant2_CentreC)
	Mandatory outdoor play	“Yes, but I do think that the message needs to go across that while the children are doing outdoor play, there is actually something intellectual going on, because otherwise people won’t buy it. Schools wouldn’t buy it, and parents wouldn’t buy it.” (Particpant1_CentreA) “They [parents] don’t take to it [bad weather] very well at all.” (Participant3_CentreA)
	Mobile play equipment	“So I think when they can move things around and make stuff out of it, the concentration and the confidence is there, isn’t it, and they’ll stay for longer, and they’ll work co-operatively as well.” (Particpant2_CentreA)
Programme design and implementation	Collaboration between experts and practitioners	“I mean, you couldn’t just have academics... I mean, that just doesn’t work, so you’d have to have people who were trying to design a programme going and watching groups as well initially, and then discussing and talking, and discussing what would work, saying what your aims and objectives are, and how to reach them, yes.” (Participant3_CentreC)
	Buy in from centre staff	“Staff need to be confident, and believe in what they are delivering, because if you go to a group, and the teacher or whoever it is, is sort of half-hearted and doesn’t believe it themselves, then it’s not going to be successful. So you need to be sort of confident, and believe in what you’re doing as well.” (Participant1_CentreD)
	Primary goal(s) for intervention	“I think a primary goal would be as well, making the parents and the kids, making them educated, making parents the educators. They’re ultimately responsible for their children’s physical development.” (Participant2_CentreD)
	Intervention delivery	“Centre staff, I think are going to be capable of doing it [delivering an intervention], as long as they’ve had the training and everything behind it, yes. I think staff’d be more confident to do it if they’ve got the training and the package and everything behind it.” (Particpant1_CentreA) “I would like it to be so many weeks of say, ourselves doing it, and then a guest coming in to do it, you know, somebody come in.” (Particpant3_CentreA)
	Duration and dosage of intervention	“I think it should just be ongoing.” (Participant4_CentreD)
	Activities and experiences	“Maybe making games and stuff out of everyday materials, or something like that, so it’s affordable for parents as well.” (Participant4_CentreC)
	Outside of the preschool	“And they [children] learn through parents as role models as well, so it’s actually engaging. There’s no point doing loads of activities just for children, if the parents aren’t actually doing the activity as well, and the children don’t follow.” (Particpant3_CentreA)
	Unlimited budget	“If we had an unlimited budget, well, we’d have a fabulous outdoor play area.” (Particpant2_CentreA)
Training for practitioners	Early Years Teacher Training	“It all comes down to training and education, because if you’ve got staff who don’t realise, if they’ve never had the early education, the pre-school learning, then they’ll go, ‘Oh yes, just give them a ball’, and that’s it.” (Participant1_CentreD)
	Understanding of physical literacy	“If we’re professionals, and we don’t understand [physical literacy], and if a parent said, ‘Oh, I don’t know what that is.’ Well, neither do we, sorry. We’ve had no training on it.” (Particpant2_CentreB) “I think there’d have to be a really big push on it [physical literacy], because, I mean, wording’s so important, and language is so important.” (Particpant1_CentreB)
	Understanding of physical activity	“Because it’s always numeracy, and whenever we go on courses, the first thing is communication and language, and maths, or literacy. It never is about being physical.” (Particpant3_CentreA)
	Child development and motor skill competence	“Because people have expectations that are unrealistic of some children, and if the children are, the difference between a child who’s maybe just two, and a child who’s nearly three, in that same room, it’s massive. So they need to be aware of child development, which a lot of them are.” (Particpant2_CentreA) “I think it’d be a good idea, but I think you’d need to look at what the skilled staff already have. We would have a good knowledge as nursery nurses, of a lot of that.” (Participant2_CentreB)“Maybe educate them [centre staff] also about certain kind of activities, what it does to children, what it does to them, because every activity’s different again, and there’s so many.” (Participant4_CentreC) “Yes. Well, you need to know what a child should be doing at a certain age. That’s it, isn’t it? You need to know where they should be.” (Participant5_CentreC)
	Training delivery	“I think you need a variety of approaches. Sometimes it’s good to have in-house training, but it’s also good to go out and see what happens in other areas, and other settings, and pick your best. Teachers are very good at that, taking all the good ideas from other places.” (Participant2_CentreC)
